# Southern Medical College Commencement

**Published:** 1882-03-20

**Authors:** 


### EDITORIALS AND MISCELLANEOUS.

### *SOUTHERN MEDICAL COLLEGE COMMENCEMENT.*

The third Annual Commencement of this institution took place at DeGive's opera house, Atlanta, Georgia, on the evening of March the 1st, 1882. The audience was very large, and composed of the best citizens of the city. The ladies were out in unusual numbers, presenting a splended array of life and beauty; the music was exquisite, and the entire scene one of rare interest and attraction.

The class of graduates was arrayed in two divisions upon the stage with the Faculty in the centre. The exercises were opened with a fervent and eloquent prayer by the Rev. Henry McDonnald.

### DEAN'S REPORT.

Dr. W. P. Nicolson, Dean, read his annual report to the Board of Trustees, touching the prospects of the Institution, in which was stated that the session now closing had been more prosperous financially and otherwise, than any previous one, giving great promise of the fulfillment of the designs of the founders, in establishing a great, leading, central medical school in Atlanta. The Institution is now well known over the entire country. The number of matriculates the present session was 124, representing the following States: Georgia, Alabama, South Carolina, Florida, Tennessee, Mississippi, Virginia, Maine, Texas and Arkansas.

The report alluded to the Hospital enterprise which had been inaugurated by the Ladies' Hospital Association and a house and lot purchased and donated to the Southern Medical College, and which failed to be in readiness for the present session as had been promised, on account of a bill of injunction filed by a few citizens in the immediate vicinity of the hospital building who, though friends to the college and the hospital enterprise, objected to its being in their immediate proximity. The injunction was successfully resisted by the Hospital Association and the Faculty and set aside, but too late to put the hospital in full operation for the present session. The hospital building is now undergoing thorough repairs, and the hospital will be in full operation on or before the first of September next, and the profession may rest assured that no effort will be spared to push forward every improvement and facility essential to the demands of the highest medical education.

### CONFERRING DEGREES.

Rev. A. J. Battle, LL. D., president of Mercer University, and a trustee of the Southern Medical College, then conferred the degree of Doctor of Medicine upon the following graduates, to-wit:

*Georgia*—J. C. Beauchamp, L. T. Boatright, J. D. Bowers, L. H. Cartledge, G. W. Clower, M. W. Coffee, J. B. Courson, G. W. de LaPerriere, I. G. Dorris, J. A. Price, F. A. Rauschenberg, J. T. Roan, W. F. Robertson, G. L. Sawyer, W. H. H. Peek, C. B. Sewell, J. H. Sims, J. N. Smith, G. C. Spearman, W. D. Vinson, M. H. White, B. T. Wise, S. J. Ellis, J. P. Gwyn, I. N. Huffaker, A. R. Jones, Dock Long, R. B. McCants, T. P. McElreath. H. F. Coleman, G. E. Jones, of Alabama. T. M. Beaty, J. W. Mitchell, of South Carolina. J. H. Young, of Virginia. W. T. Foute, of Tennessee. W. J. Hannah, of Florida. B. F. Bradbury, of Maine.

### AD EUNDEM LIST.

G. M. McDowell, Robert Harper, Pleasant Wilson, C. S. Strother, of Georgia; William Hawkins, C. S. Webb, J. M. Hundley, of Virginia; F. A. Bizell, J. H. Banks, of Mississippi; O. N. Bradbury, of Maine.

The graduates came forward separately as they were called, gracefully bowing as they received their diplomas at the hands of the Dean. During this exercise the audience was greatly animated, and numerous bouquets were showered upon the happy recipients, and rounds of applause given, the ladies participating.

### ANNUAL ADDRESS.

Dr. Battle having first formally conferred the degrees in Latin, then addressed the audience extemporaneously for a half hour in an easy, graceful and eloquent manner. He alluded first to the rapid development and remarkable success of the Southern Medical College, passing high compliments upon the Faculty. He spoke in glowing and eloquent terms of the nobility and high mission of the medical profession. Medicine, as a study, embraced many collateral branches of science—physics, chemistry, geology and astronomy constitute the ground work and basis of the profession, and when these with psycological science were conjoined, then the grandest results were to be expected. Gladstone truly said that medicine will be the profession of the future. The Doctor's remarks upon the beneficence of the profession, and its good deeds, scarcely second to the ministry, and like to that of the great Physician Himself, who went about doing good, were truly eloquent, and well calculated to impress his hearers with new and exalted views of the beauty and nobleness of the medical profession. His remarks upon the importance of cultivating a love for science and for all truth, and of keeping well up with the progress of the age, were well timed and appropriate. And his allusions to the unseen universe of knowledge, and to the God of nature, and to the fact that in honoring the profession, we should not lose sight of the Creator whom we must honor and adore as the author of all truth and of all good, were forcible, eloquent and impressive. The entire address, at which we have scarcely glanced, was among the best we have ever heard on any similar occasion, and was listened to with profound interest and attention.

### THE VALEDICTORY.

The address of the Valedictorian of the class, Dr. J. H. Sims, was then delivered, in which the young man did himself much credit. He spoke in high terms of the ability, kindness and indomitable energy of the Faculty; of their high-toned, honorable and ethical methods of conducting the school; of the deserved success and growing prospects of the Institution, and of the duty of the Southern profession and of Southern students to patronize the Southern Medical College. His farewell remarks to the class were touching, and his praise of Atlanta and her citizens invoked the hearty applause of the audience.

### AWARD OF PRIZES.

After the valedictory the Dean presented the Faculty prize of $75.00 in gold for the highest proficiency at the final examination, in all the departments, which was awarded to Dr. B. F. Bradbury, of Maine.

The second prize of $25.00 in gold was awarded to Dr. John H. Young, of Virginia.

Honorable mention was made, in this connection, of Dr. Geo. C Spearman, of Georgia. The contest between these three was exceedingly close.

Prizes were then awarded by the several Professors for the best examinations in their several branches, as follows:

By Professor Nicolson—A case of instruments to Dr. B. F. Bradbury, for the best examination in Anatomy.

By Professor Hobbs—An oppthalmoscope to Dr. Geo. C. Spearman for the best examination in Diseases of the Eye, Ear and Throat.

By Professor Roy—drug case to Dr. Geo. C. Spearman for the best examination in Materia Medica.

By Professor Johnson—A case of Surgical Instruments to Dr. John H. Young for the best examination in the Theory and Practice of Surgery.

By Professor Bizzell—An apparatus for testing urine to Dr. John H. Young for the best examination in Chemistry.

By Professor Crawford—A gold medal to Dr. J. C. Beauchamp for the best examination in Clinical and Operative Surgery.

By Professor Owens—A drug case to Dr. Geo. C. Spearman for the best examination in the Principles and Practice of Medicine.

By Professor Word—A drug case to Dr. L. H. Cartledge for the best examination in Physiology.

By Professor Powell—A gold medal to Dr. John H. Young for the best examination in Obstetrics and Diseases of Women and Children.

Each Professor, on presenting his prize, made a short and appropriate address to the recipient, which added greatly to the interest and variety of the occasion.

The benediction, pronounced by the Rev. T. R. Kendall, concluded the programme.

The impression made by the exercises was highly favorable to the present standing and future growth and prospects of this new and rapidly rising Institution.

